# CNVs into the wild: screening the genomes of conifer trees (*Picea spp.*) reveals fewer gene copy number variations in hybrids and links to adaptation

**DOI:** 10.1186/s12864-016-3458-8

**Published:** 2017-01-18

**Authors:** Julien Prunier, Sébastien Caron, John MacKay

**Affiliations:** 1 Institute for System and Integrative Biology (IBIS), Université Laval, Quebec, QC G1V 0A6 Canada; 2 Centre for Forest Research, Université Laval, Quebec, QC G1V 0A6 Canada; 3 Department of Plant Sciences, University of Oxford, Oxford, OX1 3RB UK

**Keywords:** Copy number variation, Comparative genomic hybridization, Non-model species, Genomic structural variation, Genome architecture, Species hybridization, *Picea glauca*, *Picea mariana*, *Picea Engelmanni*, conifers

## Abstract

**Background:**

Copy number variations (CNVs) have been linked to different phenotypes in human, including many diseases. A genome-scale understanding of CNVs is available in a few plants but none are wild species, leaving a knowledge gap regarding their genome biology and evolutionary role. We developed a reliable CNV detection method for species lacking contiguous reference genome. We selected multiple probes within 14,078 gene sequences and developed comparative genome hybridization on arrays. Gene CNVs were assessed in three full-sib families from species with 20 Gb genomes, i.e., white and black spruce, and interior spruce - a natural hybrid.

**Results:**

We discovered hundreds of gene CNVs in each species, 3612 in total, which were enriched in functions related to stress and defense responses and narrow expression profiles, indicating a potential role in adaptation. The number of shared CNVs was in accordance with the degree of relatedness between individuals and species. The genetically mapped subset of these genes showed a wide distribution across the genome, implying numerous structural variations. The hybrid family presented significantly fewer CNVs, suggesting that the admixture of two species within one genome reduces the occurrence of CNVs.

**Conclusions:**

The approach we developed is of particular interest in non-model species lacking a reference genome. Our findings point to a role for CNVs in adaptation. Their reduced abundance in the hybrid may limit genetic variability and evolvability of hybrids. We propose that CNVs make a qualitatively distinct contribution to adaptation which could be important for short term change.

**Electronic supplementary material:**

The online version of this article (doi:10.1186/s12864-016-3458-8) contains supplementary material, which is available to authorized users.

## Background

Studies of genetic variation have long focused on SNPs but over the last decade, novel insights into the genomic bases of phenotypic variation have come from analysing other types of polymorphisms including structural variations (SV). Genes with variable copy numbers have long been known in humans (e.g., [1]) but screening the entire genome for quantitatively variable DNA segments is more recent (e.g., [2, 3]). DNA copy number variations (CNVs) are a class of SV which may vary in size and are being intensively studied in the human as they are associated with a variety of phenotypes, mostly diseases [4].

Although, there is no strict consensus regarding the definition of CNVs, a few common features can be found. CNVs are typically described as DNA fragments exceeding 1 kb [3, 5, 6] with a minimum sequence identity of 90% [5] and most authors indicate that CNVs should not include transposons nor arise from transposon activity [7]. We refer to presence/absence variation (PAV) when zero copies are present in an individual genome [8], representing a subset of CNVs.

CNVs can either be inherited from the previous generations or appear *de novo* in an individual. The per-locus rate for genomic rearrangements was estimated between 2 × 10e^−5^ and 1.25 × 10e^−4^ in humans [4]. Mechanisms that have been identified as resulting in CNVs (for review, see [9]) include non-allelic homologous recombination [10], non-homologous end joining [11], break induced replication [12], single strand annealing [13], breakage-fusion-bridge [14] and all replication slippages of the DNA-polymerase during DNA replication and reparation [15, 16]. Despite this variety of mechanisms, the majority of CNVs are linked to the occurrence of low-copy-repeats (LCRs) that are DNA segments larger than 1 Kb, dispersed throughout the genome and share a sequence identity higher than 95%. LCRs have been reported to favor genomic rearrangements in flanking regions and described as being responsible for CNV ‘hotspots’ [17].

Gene CNVs in human have been linked to various phenotypes such as the ability to digest starch [18] and to the predisposition to diseases such breast cancer [19], among others. In plants, a SV affecting three genes was linked to pathogen resistance in soybean [20] and a gene duplication was shown to cause higher grain yield in rice [21]. Studying CNV at the genome level can even reveal evolutionary footprints, as within the primate lineage [22], which implies that inherited CNVs are subject to the same evolutionary forces as all other DNA polymorphisms. Taken together, these examples illustrate the importance of CNVs for phenotypic variation and the evolution. However, few studies have investigated CNVs and associated genes at the genome-scale in plants except for a few model species including maize [8, 23], Arabidopsis [24], soybean [25], barley [26], rice [27] and poplar [28].

A widely used approach for the discovery and analysis of CNVs at the genome scale involves comparative hybridizations on arrays of BACs or oligonucleotide sequences [2, 6, 8, 25, 29]. The method is known as aCGH (array Comparative Genome Hybridization). Oligonucleotide arrays afford rapid synthesis [29] and design flexibility, which can be advantageous to overcome the more variable signal of oligonucleotide probes compared to BACs, e.g., by using a minimum of three probes per genomic region to reduce variance [29]. As the technology has developed, statistical approaches to detect probe sets with abnormal intensities have also been implemented, most often looking for breakpoints in intensity signal between neighboring probes [29–31]. A disadvantage of these methods is that they require a high quality reference genome for the species under investigation which is often lacking for non-model organisms.

To date, gene CNVs have not been investigated in genomes as large as those encountered in most conifers (18 to 34 Gb). In this report, we developed an aCGH for white spruce (~20Gb; *Picea glauca*) and investigated North-American spruce species. White spruce and black spruce (*Picea mariana*) are largely sympatric and span from the Atlantic to the Pacific coasts in Canada. *Picea glauca* can hybridize with *Picea Engelmanni* (Additional file 1: Figure S1) to produce a hybrid known as interior spruce which is widely distributed in the Rocky Mountains [32]. Genome draft assemblies have been obtained for the diploid genomes of white spruce [33, 34] and Norway spruce [35] but are highly fragmented. However, considerable genome resources are available for white spruce including a gene catalog [36], sequence capture datasets [37], and a gene expression database [38].

In this report, we took advantage of this genomic knowledge to pursue three objectives: 1) to develop a CGH array for spruce species targeting thousands of genes and a custom analysis pipeline centered on gene sequences; 2) to determine in spruce the frequency and characteristics of CNVs and genes involved in by analysing pedigreed families; and 3) to compare results from white spruce, black spruce and interior spruce. We used our CGH array in offspring-parent comparisons and were able to detect thousands of CNVs within and across spruce families. Our findings indicated a role for CNVs in adaptation and suggested that the hybrid genome of interior spruce is less dynamic in regards to CNVs.

## Results

### Detection of CNV genes using CGH array and control for false discovery rate

**Fig. 1:**
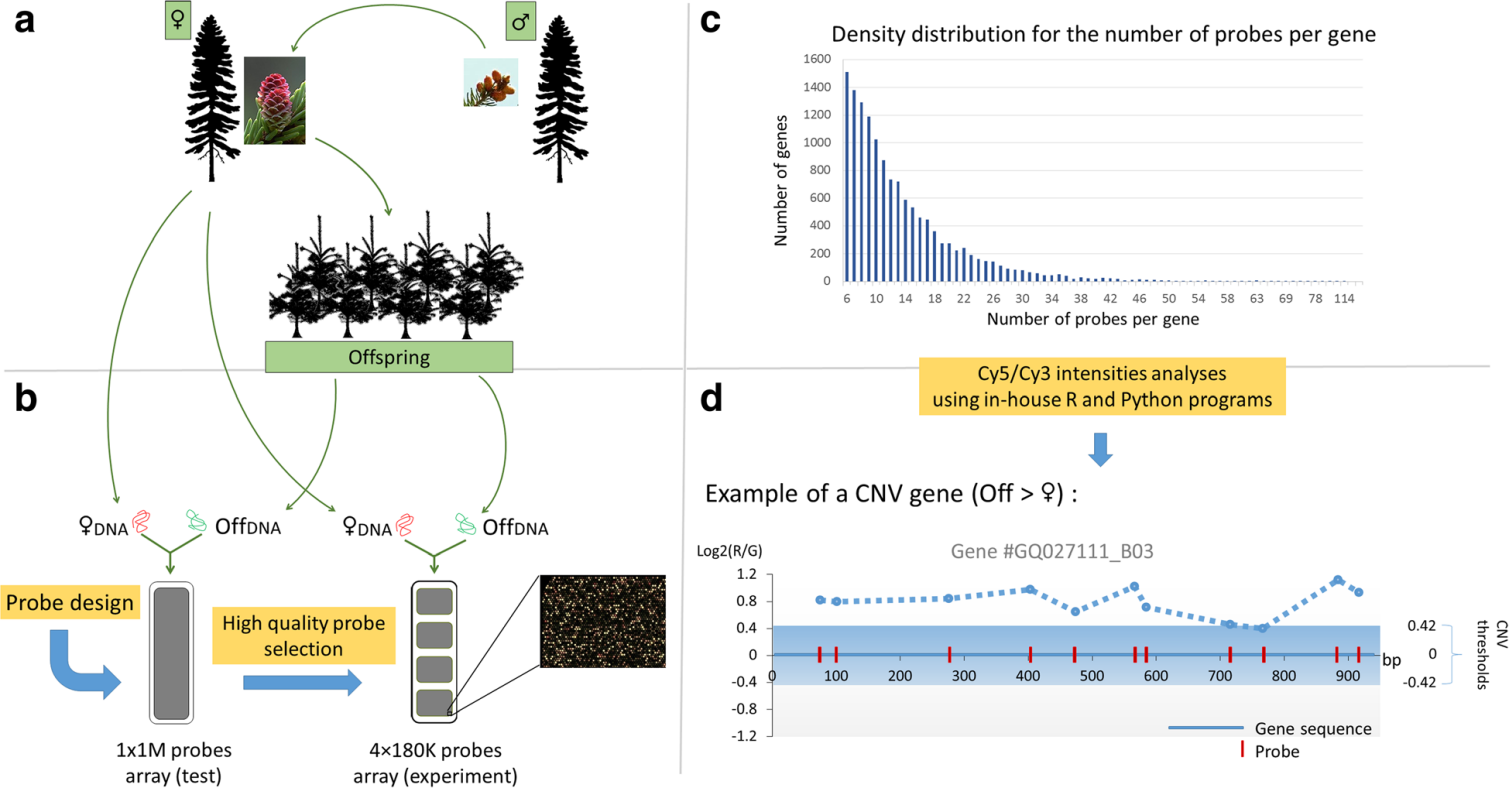
Overview of the experimental approach deployed to investigate the occurrence of CNVs within spruce pedigrees. **a**) Full-sibs are produced between two mature individuals by means of controlled cross-pollination in nursery. **b**) The ‘female’ parent genomic DNA is employed as the reference genome while the genomic DNA of descendants are used as test genomes. **c**) Distribution of probes number per gene. **d**) Distribution of probes and corrected intensity ratios along one gene sequence (GQ027111_B03) in CNV when comparing one descendant to the #77111 parent

A two-step approach was used to identify suitable aCGH probes for assessing relative gene copy number variations in comparisons between test and reference genomes (Fig. 1). We designed probes starting from a genomic assembly of the white spruce gene space which was developed from sequence-capture data and represented ~23,000 distinct genes [37] in the white spruce gene catalogue [36]. First, a set of 1 million sense and antisense probes tilled along the gene space assembly was designed by Genotypic Inc. (http://www.genotypic.co.in/) and an array was produced with the entire set of 1 million probes (Fig.1b). Second, preliminary comparative hybridizations were used to select a reduced set of genes meeting probe coverage and sequence length criteria (for details, see methods). A final set ~180,000 effective probes was used to design an array targeting 14,078 gene sequences of 500 bp or more (average 2869 bp) with a minimum of six probes (average of 12.6 probes) evenly distributed along the gene sequence; the maximum number of probes reached 114 for a single 17,259 bp gene sequence (Fig.1c).

**Fig. 2:**
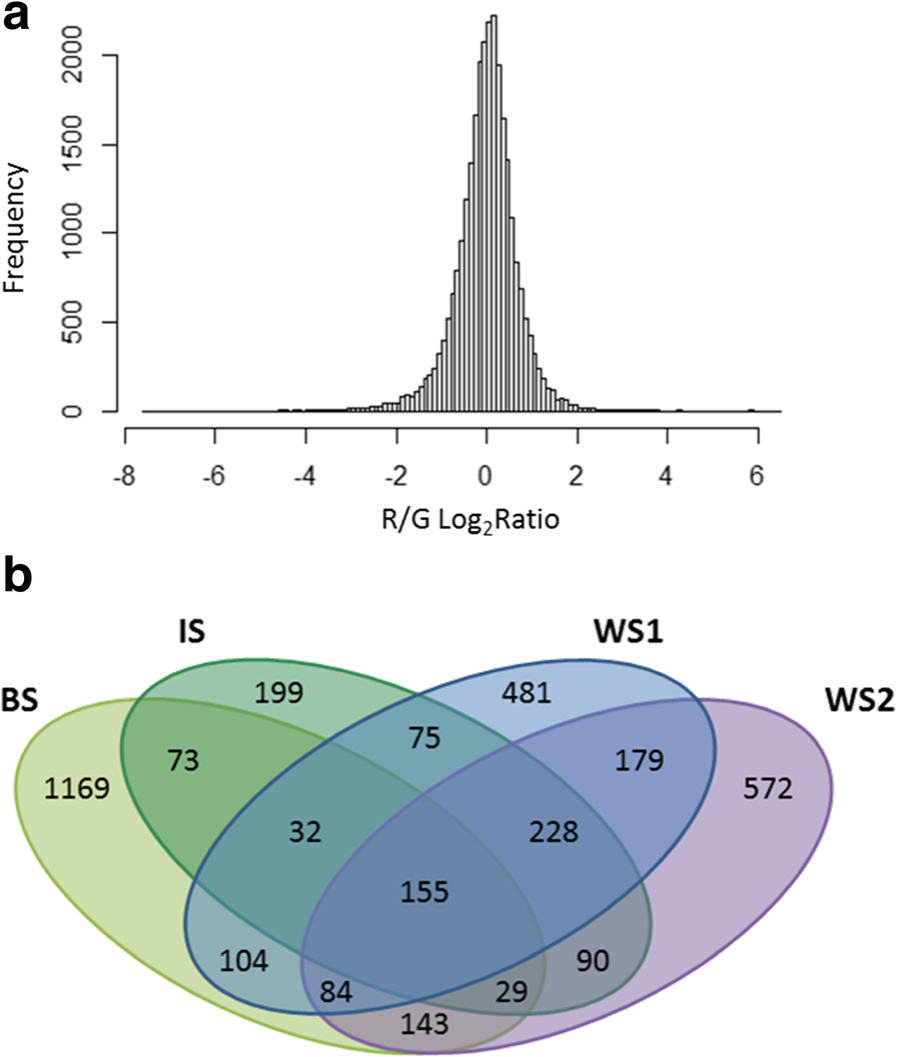
Hybridizations and CNV detection results. **a**) Distribution of the log_2_ ratios for all probes over all families. **b**) Distributions of genes found in CNV among spruce families

The 180 k probe aCGH design was effective in discovering inherited CNVs in four different spruce families by comparing the DNA of full-sib individuals (i.e., the test genomes) to one parent (i.e., the reference genome; Fig.1a-b). Here, two families were from white spruce (WS1 and WS2) and the two other families were from black spruce (BS) and interior spruce (IS, i.e., the species hybrid), respectively; we analysed 19 individuals per family in a total of 76 test-reference hybridizations. A log ratio of hybridization fluorescence intensities was calculated for each probe following the test-reference comparisons by using R package (“limma”, [39]) and in-house Python programs taking into account the dye and GC gene content biases. The vast majority of probes gave a log intensity ratio close to 0 (Fig.2a) as expected when the reference and test genomes have the same number of copies. This expected number at a given locus is two since spruces are diploid but a higher numbers of copies cannot be dismissed. Over all comparisons, the number of positive and negative ratios were very similar.

Several measures were taken to develop a reliable approach to identify genes displaying CNVs. The experimental design involved repeatedly assessing intensity ratios for a given gene by using several individuals from a pedigree and 12 probes per gene in average. Self-self hybridizations in which a single genome was hybridized in duplicate on the same array but with different dyes were used in addition to the test-reference comparisons. Self-self hybridizations were carried out in all three species (total of 9 self-self aCGH) and served to define robust CNV detection parameters, i.e., two major criteria for CNV detection, which resulted in a False Discovery Rate < < 1% in all comparisons over all species. First, an individual probe criterion was defined as: the absolute value of the log_2_ ratio must be superior to 0.42 (= |log_2_(4/3)| or |log_2_(3/4)|). Second, a gene level criterion was defined based on cross-probe repeatability such that a minimum of 83% of probes per gene must meet the log ratio criterion which translates in an average of 11 significant probes per gene declared as representing a CNV.

### Inherited CNVs were found for many different genes but with low frequencies

**Fig. 3:**
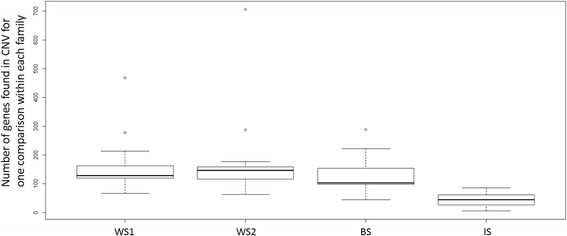
Numbers of gene CNVs within each individual for the four pedigrees. Despite some variation, the hybrid descendants presented fewer CNV genes than the pure species descendants

We detected CNVs affecting a total of 3612 distinct genes when considering all comparisons and species (Fig. 2b, Table 1). As the DNA was extracted from a sample comprising several needles (folia tissue), hence large number of cells, the CNVs most likely affected the entire tested organism and were mostly representative of a variation inherited from the previous generation. CNVs with an absolute log ratio superior to 3 made up only 2.8% to 4.0% of the gene CNVs and represented the upper limit above which large copy number differences are undistinguishable from presence/absence variations (PAVs) given the maximum intensities that can be reached using aCGH. The number of individuals scored positively for any given gene CNV ranged from 1 to 19 per family (out of 19 tested descendants). This led us to classify gene CNVs as common (i.e., in 2 individuals or more) and infrequent (i.e., in only one individual per family), the latter accounted for 64% of gene CNVs on average (Table 1). The average number of gene CNVs detected within an individual was 109.8 over all families. Two individuals in each of the white spruce families and one individual in the black spruce family accounted for nearly half of the infrequent gene CNVs in each family and represented outliers within those families (Fig. 3).

**Table 1:** Numbers of gene CNVs within each family and percentages of frequent, infrequent and presence/absence variations (PAV)

Family^a^	Number of gene CNVs	Frequent CNVs (%)	Infrequent CNVs (%)	Rate of PAV (%)
WS1	1338	36.8	63.2	3.2
WS2	1479	34.5	65.5	2.8
BS	1763	29.7	70.3	3.1
IS	881	41.4	58.6	4.4

^a^Families are named according to the main text

### CNVs were shared among species and less abundant in hybrid

The number of CNVs detected represented on average 10% of the tested genes but varied between families, i.e., 1338 to 1479 in white spruce, 1763 in black spruce and only 881 in interior spruce. The reduced number of gene CNVs in the hybrid (interior spruce) was found both at the individual level and the family level compared to the other spruce families (Table 1, Fig. 3). This difference was statistically tested and the number of gene CNVs was significantly lower in the interior spruce family than in white spruce and black spruce families (Tuckey HSD test, *P* < 1 × 10^−7^) while the difference between the three latter families was not significant (*P* > 0.69).

We examined the nature of the differences between species by considering the overlap in the gene CNV sets between the pedigrees. The different families shared from 289 to 646 gene CNVs, including a core set of 155 gene CNVs found in all spruces (Fig.2b). The number of shared gene CNVs among families was in accordance with the relative divergence between the spruce species. The two white spruce families had the most shared gene CNVs (646), white spruce and the interior spruce families had fewer (609) and the black spruce family shared the smallest numbers of gene CNVs with other pedigrees (547; Fig.2b).

### Gene CNVs are enriched for adaptation-related functions and narrow expression profiles

The potential biological implications of gene CNVs were evaluated using enrichment tests for terms in Gene Ontology annotations. In white spruce, the comparison of gene CNVs to the entire set of tested genes on the CGH array revealed a significant enrichment in a number of functions related to 16 biological processes (Table 2) most significantly including the ‘response to stress’ term. Using the recently updated PFAM annotations of the white spruce gene catalog [35], enrichment was found for oxygenase, synthetase, hydrolase and cytochrome-P450 activities. In black spruce, GO-term enrichment was found in eight biological processes including most significantly ‘response to stress’ and ‘defense response’ (Table 2).

**Table 2:** Enrichment test in GO annotations for white and black spruce species, results from FatiGO in Babelomics 4.3 (Medina et al. 2010)

Species	Term	Term description	adjusted *P*-value
*Picea glauca*	GO:0006950	Response to stress	0.0003
	GO:0006464	Protein modification process	0.0003
	GO:0006793	Phosphorus metabolic process	0.0003
	GO:0006796	Phosphate containing compund metabolic process	0.0003
	GO:0016310	Phosphorylation	0.0003
	GO:0006468	Protein aminoacid phosphorylation	0.0003
	GO:0055114	Oxidation reduction	0.0004
	GO:0043687	Post-translationnal protein modification	0.0004
	GO:0006629	Lipid metabolic process	0.0007
	GO:0006915	Apoptosis	0.0007
	GO:0012501	Programmed cell death	0.0009
	GO:0006952	Defense response	0.0018
	GO:0019748	Secondary metabolic process	0.0030
	GO:0008219	Cell death	0.0043
	GO:0042221	Response to chemical stimulus	0.0076
	GO:0006725	Cellular aromatic compound metabolic process	0.0076
*Picea mariana*	GO:0006950	Response to stress	0.0007
	GO:0006952	Defense response	0.0011
	GO:0012501	Programmed cell death	0.0016
	GO:0045087	Innate immune response	0.0016
	GO:0008219	Cell death	0.0020
	GO:0006915	Apoptosis	0.0053
	GO:0006955	Immune response	0.0055
	GO:0007154	Cell communication	0.0084

The different numbers of gene CNVs detected between species did not translate into functional annotation differences. The core set of 155 gene CNVs found in all of the families is significantly enriched in functions related to defense response. The hybrid family sharing gene CNVs with the white spruce families may be expected by inheritance of genetic material from the white spruce species but did not explain gene CNVs also shared with black spruce. Hence, such CNVs sharing between all families is more likely indicative of ancestral genetic variations that remain variable in several North-American *Picea* species. In addition to defense responses-related functions, all spruce species and the hybrid were enriched in the stress responses while annotations of the 1242 genes specifically found in black spruce were not significantly enriched in any biological process compared to white spruce specific genes. On the other hand, the 199 genes specifically found in the hybrid were not enriched in functions related to defense nor stress responses but enriched in functions related to responses to hormonal, organic and chemical substances.

Expression levels for 81.5% of the gene CNVs were previously assessed in various organs at different stages of development in white spruce for a total of eight tissues [38]. Gene CNVs appeared expressed in wide range of tissues and organs, including vegetative buds, foliage, primary and secondary xylem, phelloderm, roots, megagametophyte and embryogenic cells with no significant enrichment for any of them. However, the set of gene CNVs is significantly enriched in genes expressed in only one or two tissues, with no or little variation between tissues.

### CNVs were distributed across the entire genome

**Fig. 4:**
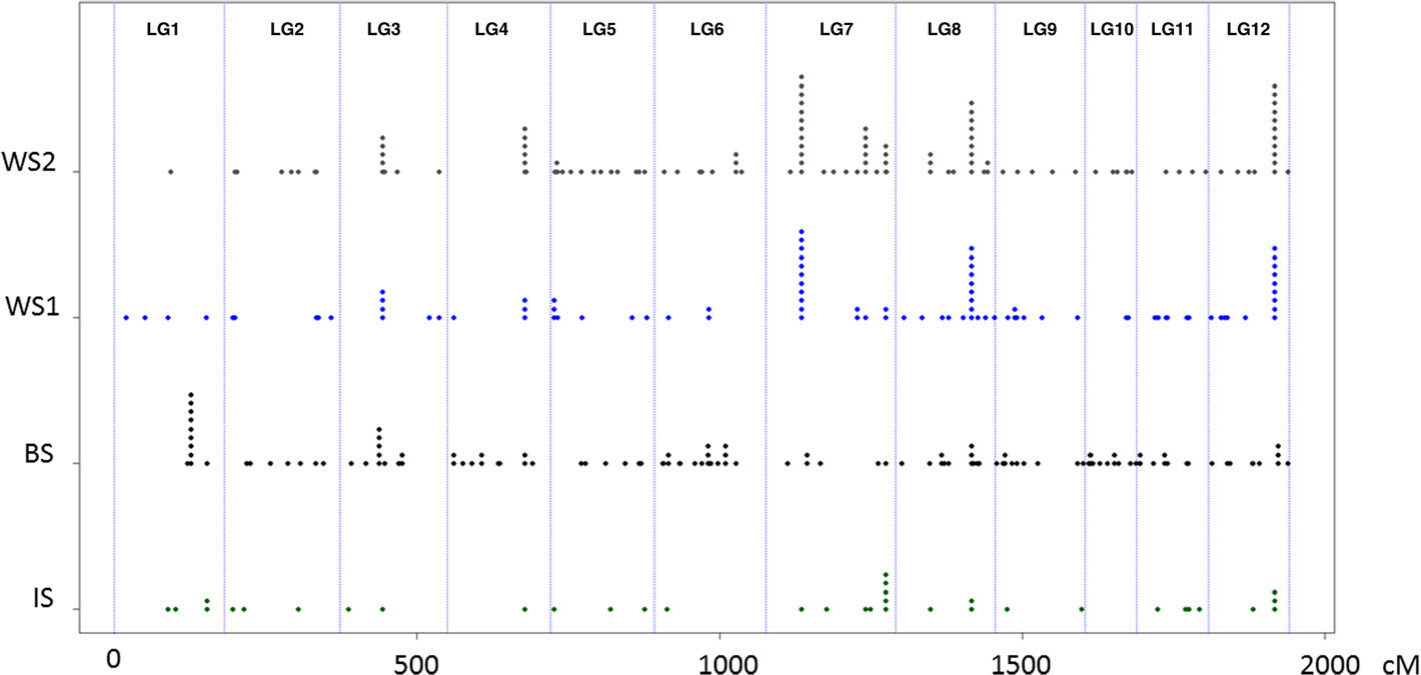
Distribution over the genome and frequency of genes found in CNV also positioned upon the spruce genetic map [40]. Each point represents the position of a gene found in CNV in one CGH comparison; the piling is indicative of the number of individuals presenting the CNV

A dense genetic map has been continuously developed since 2006 for white spruce [40–42], which has allowed to locate the position of 1766 genes, observe a high synteny between spruce species and dissect the genomic architecture for several traits of adaptive and economic interest in spruce species [43, 44]. A total of 210 (5.8%) of the CNV genes were located on this spruce genetic map. The CNVs were distributed over the entire genome; each linkage group included from 10 (LG1) to 24 (LG10) gene CNVs and had an average of 17.5 CNVs (Fig. 4). The average distances between two mapped gene CNVs were 27.26, 18.75, 16.28 and 45.26 cM for the WS1, WS2, BS and IS families respectively, and was inversely proportional to the number of mapped gene CNVs in each family. No significant clustering of gene CNVs was observed within linkage groups although a total of 10 cases of two or more genes within a 1 cM region were found across the three spruce species (Additional file 2).

## Discussion

We analysed thousands of gene CNVs and found that they are less abundant in a hybrid compared to two pure spruce species. The biological functions of the gene CNVs indicate a role in stress and defense responses. We discuss the development of CNV detection in wild species and the insights gained from our findings into genome biology, evolution and adaptation.

### Detecting gene CNVs within pedigrees

We developed gene-based CNV detection procedures to analyse wild species, even those with large genomes. A low threshold was used for CNV detection (test to reference genome log_2_ ratios > 0.42) compared to other recent studies (e.g., [8, 23, 26]). This apparently low value was permitted because of an array design that included a relatively large number of probes which presented such significant signal for the same gene. Previous reports have recommended a minimum of three probes per genomic segment in order to effectively estimate the relative number of copies when using oligonucleotide CGH (e.g., [45]). When analysing genes, some authors set a requirement that all of the three to four probes tested be significant for positive scoring of CNVs (e.g., [23]). We used many more probes per gene sequence (12.6 probes on average) which provided a strong support for the estimated ratios and robust detection parameters with very low FDR. In these conditions, the number of CNVs detected was inflated substantially when we lowered the proportion of significant probes (results not shown) and was only slightly affected when we lowered the significance threshold for intensity ratios.

We did not detect a bias towards negative or positive log ratios in the overall data set nor when comparing individual offspring to a parental reference genome. This result appeared to be at odds with those from other organisms showing that losses often dominate over gains [8]; however, the detection of more losses than gains could be due to a reference genome bias, at least in some cases [8, 23]. This type of bias is not expected in our experiment as we used parent-offspring comparisons which reduced sequence divergences between the test and reference genomes, both being similarly distant from the genome used for probe design. It has also been argued that a higher number of positive CNVs (i.e., copy gains) may be expected because genomes are likely more tolerant to duplications than deletion possibly resulting in loss of function [46].

Our approach, in which we sampled multiple offspring, was designed to uncover and identify inherited pre-existing gene CNVs shared across a pedigree rather than *de novo* CNVs arising from recent structural variations. However, many of the CNVs in spruces (50 to 66%) were found in only one individual within a pedigree, as observed in other plants [26, 46, 47]. These infrequent CNVs may represent three different cases: i) random effects due to the small sample size (19 individuals per family), which is supported by the detection of these same CNVs in at least one other family (275 cases); ii) false-positives, despite the very small number of CNVs in self-self hybridizations; iii) *de novo* structural variations which occurred in or during reproduction of the previous generation. In the subset of CNVs found only once over the entire dataset (*N* = 1919; including the four pedigrees) the proportion of negative and positive log ratios was balanced, suggesting that there was no prevalence of *de novo* copy gains or losses.

### Impact and distribution of CNVs across the genome

The proportion of genes positively scored for CNVs was 15.5% in white spruce (considering both families) which is similar or slightly higher in magnitude than found in whole genome analyses in model species and crop plants ([3] (human); [48] (mouse); [49] (rat); [50] (human and chimpanzee); [8, 23] (maize), [26] (barley)). The high proportion found in spruce can be either attributed to the detection of relatively short CNV regions enabled by the high probe density in our array design, or the occurrence of more than one gene located within the same long DNA sequence in CNV. In that case, some CNVs may represent only one and the same DNA segment that has been duplicated or lost. This could not be verified for most of the CNVs as their genomic positions were unknown. Hence, the rate of CNVs may not precisely reflect the rate of actual structural variations but rather provided an estimate of the order of magnitude.

In total, 9% of the genes surveyed (i.e., 1,284 out of 14,078) using aCGH and 6% of the genes found in CNV had previously been positioned on a genetic linkage map in *Picea glauca* [40]. This lower proportion of mapped CNVs than expected by chance alone is not surprising because the map was mainly developed using SNP from large high-throughput genotyping arrays [40] which likely selected against genes that display CNVs. Localizations on the genetic map showed that CNV genes were widely distributed at a low density over the entire genome (Fig. 4) with a minimum of 10 CNV genes per linkage group and average distance of 27 cM between successive CNVs within species. In addition, 10 cases of 2 or 3 CNV genes located within 1-cM of each other suggested the occurrence of structural variations involving chromosomal segments with more than one gene. Even though the number of mapped CNVs was too small to precisely assess the frequency of such events, it would represent an estimated rate of SVs close to 4.3%. Multi-gene SVs have already been described in soybean [20] and a rate of 5–10% was reported in maize/teosinte by using aCGH [23]. In light of these reports, more SVs affecting more than one gene in spruce could be expected. However, conifer genes are often separated by very long non-coding regions [33–35, 51] such that multi-gene SVs may be less common in this phylum. Furthermore, the discovery of CNVs in close proximity to each other raises the question of their origin. The first hypothesis may be that these genes are among the few genes actually separated by relatively small non-coding sequences. Another explanation would be that these genes are widely separated but structural variations that are typically even wider in conifers, hence allowing to encompass several coding sequences. Finally, one possible explanation is that these genes may otherwise originate from different events of SV within the same genomic region; such hotspots for structural variations have been found in other model organisms [52–54]. Further characterization of these genetically linked CNVs genes and a finer dissection of structural variations could be achieved by analysing larger populations (~100 segregating individuals), hence allowing to directly map these genetic variations and infer the occurrence of one or more events of structural variation.

### Numbers of shared CNVs agrees with the estimated time of species divergence

The proportion of shared gene CNVs across the different *Picea* species was in accordance with their phylogenetic distance, i.e., more gene CNVs were shared among more closely related families and species. The highest overlap was found between the two white spruce families, suggesting that the 646 shared gene CNVs are not limited to one parental lineage but more likely to be spread at a significant level in the population. The second highest overlaps were between interior spruce and white spruce families despite the lower number of gene CNVs in the hybrid. Engelmann and white spruces, the parental species of the hybrid interior spruce, diverged only 9.9 Myears ago, a relatively short timeframe compared to the 38.1 Myears separating the white/Engelmann spruce lineage from the black spruce lineage [55]. In line with their relative times of divergence, Engelmann and white spruce can hybridize while black spruce is reproductively isolated from both of the other species [56, 57] and also shared the smallest number of CNVs with the other spruce families.

Evolutionary forces have also been linked to CNV diversity in other plants. In a study comparing domesticated and wild barely accessions for instance, it was shown that strong directional selection associated with breeding in barley reduced CNV diversity [26]. Hence, CNV diversity in barley mirrored the behaviour of other DNA polymorphisms indicating that evolutionary forces likely have similar impacts on CNVs than on other types of DNA polymorphism. Similarly, CNV diversity assessed using a human aCGH allowed to detect gene CNVs shared among primates that likely resulted from gene duplication in common ancestors [22]. Therefore, gene CNVs shared between the different spruce species likely represent ancestral polymorphisms that have not been eliminated by genetic drift or directional selection during the course of evolution, or alternatively, have been maintained by balancing or divergent selection.

### Role of CNVs in the evolution of forest trees

A wide range of gene CNVs have been linked to quantitative phenotypic variation in model organisms [18, 20, 23, 58]. In forest trees, quantitative genetic variations have been linked to adaptation and is characteristic of key adaptive traits such as growth phenology and cold tolerance. Genetic variation in adaptive traits has been studied in many conifers including *Picea spp.* and was often observed to follow gradual changes over wide ranging geographic areas and environmental conditions as opposed to drastic changes linked to specific conditions [59–61]. Here we examine the potential impact of CNVs on adaptation in trees and predict that they make a qualitatively distinct contribution compared to variations in coding sequences resulting in amino acid substitutions.

The two types of mutation have different impacts at the molecular level and are expected to have different evolutionary consequences. Changes in amino acid sequences may alter protein function and therefore are more often likely to be deleterious and purged, or to be recessive and only impact phenotype in homozygotes [62]. CNVs alter gene dosage and may affect expression which is akin to cis- acting regulatory variation. As such CNVs are more likely to alter phenotype in heterozygotes and thus be more directly visible to selection [63]. Gene dosage effects caused by CNVs may also be detrimental, for example by creating an imbalance in multi-protein complexes [62], but this is likely to be restricted to certain classes of genes and be more prevalent in house-keeping functions.

The relative effects of CNVs and amino acid changes will also depend on the function and expression of the genes product. Here, CNV genes were enriched for biological functions related to stress response, defense response and metabolic processes, which was also observed in barley and maize [23, 26] and is in line with a role in adaptation. The CNV genes were also enriched for narrow transcript accumulation profiles in white spruce [38], suggesting more specialized functions rather than house-keeping roles which reinforces the potential for CNVs to contribute to adaptive phenotypes. Considering these observations, CNVs affecting genes have the potential to make a unique contribution to adaptation in trees as a result of their qualitatively distinct impact on evolution and the types of genes affected. We further suggest that CNVs could be especially important for short term adaptability and thus in responding to the acceleration of environmental change.

### Fewer CNVs in the hybrid species

Significantly fewer CNV genes were found for the interior spruce family (IS), a hybrid resulting from the natural introgression of white and Engelmann spruce. We examined whether the difference could be due to technical reasons such as IS producing fewer successful hybridizations as the probe set was designed with pure white spruce sequences. However, the data indicated that the numbers of successfully tested genes were nearly identical in all three species including the more distantly related black spruce. This is consistent with the expectation that the procedure we used was able to tolerate up to three mismatches per probe (Genotypic Inc. *pers. comm.*) and the average degree of polymorphism found among sequences of white, black and Engelmann spruces.

The occurrence of CNVs is underpinned by mechanisms that are likely to be conserved across all living organisms [9] and are predominantly associated with low-copy-repeats (LCRs), such as unequal interchromosomal recombinations, among others. LCRs are conserved (identity > 95%) DNA segments that occur a few times throughout the genome [9]. Interior spruce has a diploid genome that originally came from contributions of white spruce and Engelmann spruce. As a result, a higher level of heterozygosity is observed in the individuals of interior spruce compared to individuals of pure species. Such dual origin is expected to have introduced a higher level of diversity in LCRs found in hybrid than that found in individuals of pure species. Hence, a lower sequence identity between LCR sequences of homologous chromosomes is expected in the hybrid genome which would have entangled the mechanisms leading to gene CNVs. As a consequence, the interior spruce individuals has less numerous CNVs than white spruce or black spruce individuals; these CNVs may result from other types of breakage-reparation mechanisms that are not based upon sequence identity. These results suggest that hybrids have systemically reduced copy number variability indicative of a less dynamic genome which in turn would limit genome evolvability. Future studies investigating other cases of introgression, either from the wild or from artificial cross-breeding, would support or dismiss this hypothesis.

## Conclusion

Investigations of non-model species have broadened the scope of genomic studies and contribute to the understanding of ecological diversity. More affordable sequencing and genotyping techniques as well as the advent of Next-generation sequencing have paved the way to these developments. This report illustrates how techniques of transcriptomics and gene-space characterization in a non-model system can in turn be used to design CGH arrays and study CNVs on a genome scale. Approaches using whole genome sequencing data from model organisms to detect usually small CNVs have been developed; however different detection methods and algorithms have produced different results from the same datasets [64]. More recently, continuous development of technologies to sequence and characterize larger DNA segments have allowed to investigate larger structural variations in model organisms using third generation PacBio sequencing [65] or genome mapping on nanochannel arrays [66]. These technologies can be applied to non-model organisms, although the 20-Gb conifer genomes would still remain challenging.

An advantage of the approach outlined here was the very low FDR which indicated robust results. Although it is time and resource consuming, testing several offspring from the same parents was also helpful as it confirmed the discovery of CNVs by repeated identification in related individuals. This approach also throws light onto inherited gene CNVs which are of interest for investigating the role of CNVs in adaptation and evolution as well as establishing a framework to study their relation to phenotype, e.g., through QTL mapping. As gene CNVs could result in dosage effects on phenotypic variation, they may play a key role in quantitative trait variations such as growth and phenology in plants.

Here, we reported a lower number of CNVs within a hybrid pedigree than in families from pure species, suggesting that hybrids may systemically have a lower diversity in CNVs. Considering the gene annotations related to stress and defense responses, this reduced genetic diversity may act on phenotypic variability and evolution of hybrids in response to natural selection. Future investigations to assess the impact of CNVs on the evolution of species could focus on CNVs at the population level, looking for signatures of selection across geographical ranges and varying environmental conditions. Applied to a variety of species, such analyses may reveal new insights into adaptation and speciation.

## Methods

### Pedigrees & plant material

Full-sib trees issued from four controlled crosses were sampled in common garden sites. Two pedigrees (2516: 77111 × 2388; 2856: 80109 × 80112) were sampled in the Quebec province (East Canada, supporting Additional file 1: Figure S1) and represent pure white spruce trees that were already investigated and genotyped for genetic mapping and QTL mapping of adaptive traits and growth [41, 43]. The third pedigree (Pg29 × Pg79) was sampled in British-Columbia (West Canada, Additional file 1: Figure S1) and represents an introgressed progeny of white spruce and Engelmann spruce. Finally, the fourth pedigree (Pm1442 × Pm1425) was sampled in the North of the species distribution in the Quebec province (East Canada, Additional file 1: Figure S1), remotely from the sympatric zone between *Picea mariana* and *Picea rubens*, hence represent pure black spruce trees.

### DNA extraction

Whole shoot tips composed of needles, buds and apex were collected during early spring and kept at minus 80 °C until further use. Tissues were ground to a fine powder using a MixerMill MM 300 (Retsch, http://www.retsch.com/). Genomic DNA (gDNA) was extracted with DNeasy Plant Mini Kit (QIAgen, http://www.qiagen.com/). DNA concentration was determined using a NanoDrop 1000 (Thermo Scientific, http://www.thermoscientific.com/). Quality was assessed by agarose gel electrophoresis (1%, TAE buffer) of 100 ng gDNA per sample.

### Marking, hybridization and visualizing

Array CGH hybridizations were performed with the SureTag DNA Labeling Kit (Agilent, protocol version 7.2), following manufacturer’s instructions using the manual configuration for the 1 × 1 M array and the Tecan configuration for the 4 × 180 K array. HS400 Pro hybridization stations (Tecan, http://www.tecan.com/) were used with the Tecan configuration.

### Probe and array designs from genome capture

A sequence capture assembly is available for white spruce since 2014 [37]. For this work, custom Nimblegen probes were designed from a gene catalog for white spruce [36] and included in a chip targeting 23,864 genes. The captured DNA was sequenced using 454 technology and sequences were assembled using the gsAssembler module of Newbler (v2.53). A total of 83,362 contigs (55.87 Mb total length) were obtained, only those corresponding to known *Picea glauca* trancripts were considered, representing ~23,000 genes and including mostly exons and introns < 1Kbp.

Probe design was realized by Genotypic Inc. (http://www.genotypic.co.in/) using these genomic sequence data after filtering out highly repeated sequences. Probes were designed in sense and antisense orientation based on GC content, Tm and PolyX, with an average probe tiling of 30 bp. Probes were validated according to these criteria: single hit against the target, alignment length of 60 bp, less than 3 allowed mismatches and less than 2 allowed gaps. After validation, cross-hybridizing probes were removed. In the end, 3,661 contigs were removed from sequence data because probes did not pass criteria. 971,042 probes were kept for the final design, representing 22,219 *Picea glauca* genes [36].

Final design was sent to Agilent (Agilent Technologies, http://www.agilent.com/) through their SureDesign platform (https://earray.chem.agilent.com/suredesign/) for printing on SurePrint G3 Custom CGH Microarrays 1x1M. This first CGH microarray was tested 5 times using the individuals from the different crosses, comparing the gDNA from offspring to the gDNA of its parent.

These comparisons were analyzed by Genotypic who selected high quality probes according to the following criteria. Raw intensity had to be twice background intensity in at least 6 datasets out of 10 (5 hybs × 2 channels). Sense or antisense probe targeting the same position were removed based on lowest average intensity across the 10 datasets. There had to be a minimum 6 probes per gene. The length covered (i.e., distance between start of first probe and end of last probe) had to be more than 500 bp, otherwise the gene was not considered. Probe distribution across contigs was adjusted with minimum spacing of 200 bp. However if number of probes per transcript was less than 16, spacing was reduced to 100 bp.

The final number of probes on the array is 177 223, representing 14,078 transcripts. Final design was sent to Agilent through the SureDesign platform for printing on SurePrint G3 Custom CGH Microarrays 4x180K.

### Data treatment

The fluorescence intensities were analyzed using an in-house pipeline of R (v3.1.3) and Pyton (v2.7.9) scripts (available in https://bitbucket.org/jprunier/git-CNVgene-discovery) in order to correct for dye bias, take into account the GC content in the intensity variations and finally compare the ‘test’ genome (a descendant) to the ‘reference’ genome (a parent).

To take into account the technical variability among slides and cytochromes, a LOESS correction was performed using the ‘limma’ package in the R distribution [38]. Intensity ratios were normalized applying the robust LOESS regression on the M-A plot, where M = log_2_(I1/I2) and A = log_2_[(I1 _ I2)1/2], with I1 and I2 being the background subtracted intensities of the spot in the two images.

In addition, given the well-known effect of GC content on the intensities, also called “genome waves” [67], the effect of the GC content of each contig on the signal intensity was tested and correction was made to take into account such effect. In line with this, the effect of the bait (=probe) composition was also highlighted as a possible biasing factor in arrays. Hence, the composition effect on signal intensities was also tested and the intensities ratio was adjusted accordingly for each probe.

Our dataset presented an originality since it presented an average number of 12.6 probes per gene, in other words 12.6 probes per DNA regions. Thus, two parameters could be explored, i.e., the minimum absolute intensity ratio to consider a probe significant and the proportion of probes presenting such ratio to consider the gene in significantly different copy numbers between the test and reference samples. In order to delineate a set of parameters allowing to robustly identify gene CNVs, we tested series of detection thresholds and proportion of significant probes to analyze self-self hybridizations and assess the False Discovery Rate (FDR). These self-self hybridizations were performed for the ‘reference’ genomes of WS1, BS and IS families, for a total of 9 self-self aCGH tests thus covering all species and hybrid. An absolute value of the log_2_ ratio of 0.42 corresponding to a variation of three over four copies of the gene (|log_2_(3/4)| = |log_2_(4/3)|) as a significant threshold for each probe and a proportion of 0.85 significant probes per genes resulted in a very low FDR (<<1%).

All tested genes (14,078) were annotated according to PFAM and the Gene Ontology for homologous sequence in TAIR [36] and all enrichment tests for genes found in CNV were performed using FatiGO in Babelomics 4.3 (http://v4.babelomics.org/functional.html) [68]. Gene genomic locations were obtained from the spruce genetic map [40] and the density distributions over the genetic map for gene CNVs and all mapped genes were compared by means of Kolmogorov-Smirnov test using the ‘ks.test’ function in R (v3.1.3).

## Additional files

Additional file 1: Figure S1. Map of parent provenances for the four pedigrees. (DOCX 240 kb)
Additional file 2: Table S1. Distribution of the gene CNVs along the spruce genetic map with position in cM. (XLSX 15 kb)

## Abbreviations

aCGH: Comparative Genomic Hybridization on array
CNV: Copy Number Variation
PAV: Presence/Absence Variation
QTL: Quantitative Trait Loci
SV: Structural Variation
