# Supplementary material for: CNVs into the wild: screening the genomes of conifer trees (Picea spp.) reveals fewer gene copy number variations in hybrids and links to adaptation
Source: BMC Genomics. 2017 Jan 18;18:97. doi: 10.1186/s12864-016-3458-8 (PMC5241962; doi:10.1186/s12864-016-3458-8)
Supplement: Additional file 1: Figure S1. — Map of parent provenances for the four pedigrees. (DOCX 240 kb) [file 12864_2016_3458_MOESM1_ESM.docx]

Additional file 1: Figure S1


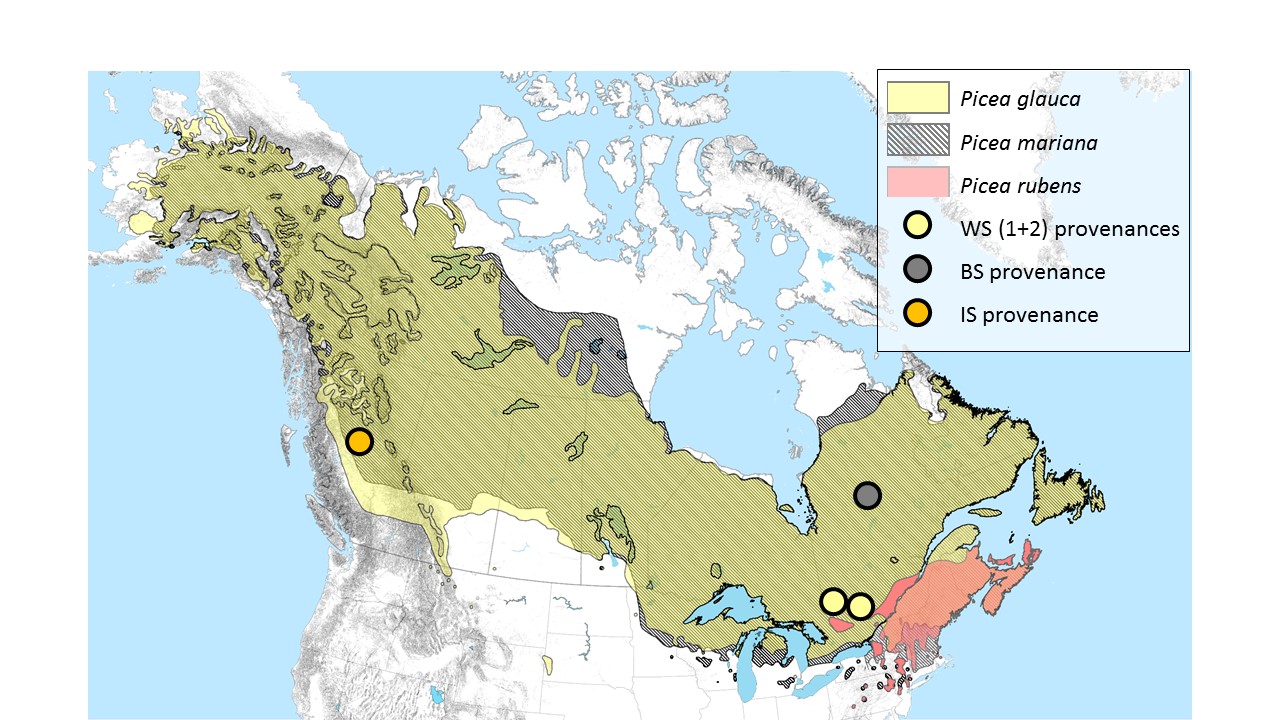


Species distribution and provenances for the different tested pedigrees: WS1, WS2, BS and IS (see main document for description).
